# Impact of Lipid Metabolism on Macrophage Polarization: Implications for Inflammation and Tumor Immunity

**DOI:** 10.3390/ijms241512032

**Published:** 2023-07-27

**Authors:** Evros Vassiliou, Renalison Farias-Pereira

**Affiliations:** 1 Department of Biological Sciences, Kean University, Union, NJ 07083, USA; evassili@kean.edu; 2 Department of Plant Biology, School of Environmental and Biological Sciences, Rutgers, The State University of New Jersey, New Brunswick, NJ 08901, USA

**Keywords:** cancer, inflammation, lipid, macrophage, metabolism, polarization

## Abstract

Macrophage polarization is influenced by lipids, which also exert significant control over macrophage functions. Lipids and their metabolites are players in intricate signaling pathways that modulate macrophages’ responses to pathogens, phagocytosis, ferroptosis, and inflammation. This review focuses on lipid metabolism and macrophage functions and addresses potential molecular targets for the treatment of macrophage-related diseases. While lipogenesis is crucial for lipid accumulation and phagocytosis in M1 macrophages, M2 macrophages likely rely on fatty acid β-oxidation to utilize fatty acids as their primary energy source. Cholesterol metabolism, regulated by factors such as SREBPs, PPARs, and LXRs, is associated with the cholesterol efflux capacity and the formation of foam cells (M2-like macrophages). Foam cells, which are targets for atherosclerosis, are associated with an increase in inflammatory cytokines. Lipolysis and fatty acid uptake markers, such as CD36, also contribute to the production of cytokines. Enhancing the immune system through the inhibition of lipid-metabolism-related factors can potentially serve as a targeted approach against tumor cells. Cyclooxygenase inhibitors, which block the conversion of arachidonic acid into various inflammatory mediators, influence macrophage polarization and have generated attention in cancer research.

## 1. Introduction

Lipids were initially recognized as structural components of cellular, organelle, and nuclear membranes. Recently, lipids and their metabolites have been increasingly acknowledged as key players in intricate signaling pathways that modulate macrophages in multiple ways, including modulating their response to pathogens, phagocytosis, and inflammation. Their signaling properties, by utilizing metabolic mediators and transcription factors, can affect cytokine expression in multiple ways [1,2]. Thus, understanding how lipid metabolism can regulate macrophage behavior may be useful for potential therapies.

Lipid metabolism can be classified into different anabolic and catabolic pathways, which include lipogenesis, fatty acid β-oxidation, lipolysis, and lipid uptake and transport. The different classes of lipids, such as cholesterols, phospholipids, and triglycerides, have distinct roles in macrophages. For example, cholesterol levels are important for the regulation of lipogenesis and inflammation [3,4]. The metabolism of other lipids (e.g., triglycerides, phospholipids, and fatty acids) also has an important role in macrophage functions, which is the focus of this review.

## 2. Macrophage Polarization

Macrophage polarization is a classification term used to describe the immunological properties of macrophages in terms of two broad categories, M1 and M2 [5]. M1 macrophages are inflammatory in nature, are typically considered anti-tumor cells, and are able to augment immune checkpoint blockade treatment [6]. They are capable of killing invading species and engaging in extensive antigen presentation. Cytokines such as tumor necrosis factor alpha (TNF-α), interleukin 6 (IL-6), and interleukin 1 beta (IL-1β) and chemical species such as reactive oxygen species (ROS) and nitric oxide (NO) are characteristic of M1 macrophages [7]. On the other side of the spectrum, M2 macrophages are anti-inflammatory and produce primarily TGF-β and IL-10. They participate in tissue repair and immune resolution. While the M1/M2 classification provides a framework in which macrophages can be classified, it is becoming increasingly evident that there are many intermediate states between the two types. Others have previously reviewed the polarization and heterogeneity of macrophages in more depth [8,9]. Furthermore, there seems to be a non-static state that allows macrophages to continuously adapt depending on the prevailing conditions. The environment in which macrophages operate is among the most influential drivers of polarization. As such, lipids can have a profound effect on macrophage polarization. Among the most studied lipid moieties capable of inducing M2 polarization are oxidized low-density lipoproteins (oxLDLs) [10,11]. They have been shown to support foam cell formation and are prevalent in atherosclerotic lesions [12,13]. It is, in a way, paradoxical for an M2 phenotype to be inflammatory and lead to pathogenesis when M2 macrophages are more likely to be involved in tissue repair [14]. The metabolites of arachidonic acid, an omega-6 essential fatty acid, like prostaglandin E2 (PGE_2_), are also known to encourage an M2 phenotype [15]. Omega-3 metabolites such as resolvins and neuroprotectins favor an M2 polarization and, overall, suppress tumor growth [16]. At the transcriptional level, peroxisome-proliferator-activated receptors (PPARs) and liver X receptors (LXRs), well-known lipid-binding factors, influence macrophage polarization [17]. These factors related to lipid metabolism are components of a complex metabolic pathway that impact various functions of macrophages.

## 3. Lipid Metabolism

### 3.1. Lipogenesis

**Figure 1:**
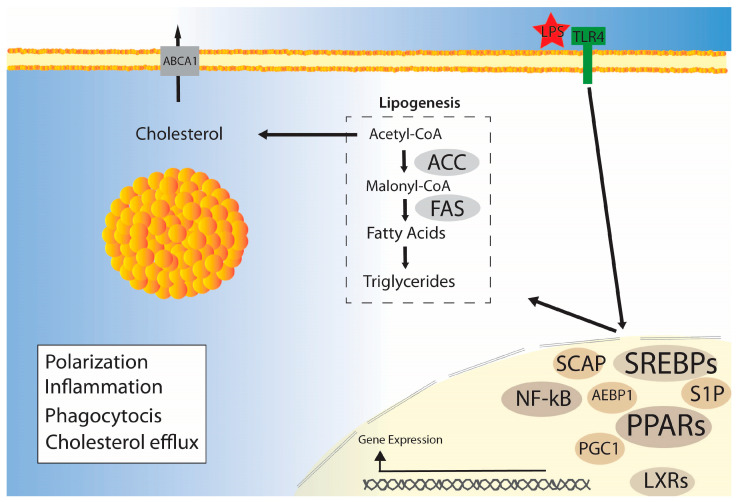
Schematic figure depicting lipogenesis and cholesterol metabolism. Acetyl-CoA can be used for the synthesis of fatty acids. ACC (acetyl-CoA carboxylase) and FAS (fatty acid synthase) are key enzymes for lipogenesis, which is enhanced in classically activated macrophages via LPSs (lipopolysaccharides) and the TLR4 (Toll-like receptor 4) pathway. Acetyl-CoA can be converted to cholesterol, which can be further esterified and included in lipid droplets as well. Several transcription factors and co-factors regulate fatty acids and cholesterol metabolism and their effects on macrophage polarization, inflammation, phagocytosis, and cholesterol efflux. Examples of these factors include SREBPs (sterol regulatory element-binding proteins), PPARs (proliferator-activated receptors), LXRs (liver X receptors), S1P (SREBP processing through site-1 protease), SCAP (SREBP cleavage-activating protein), AEBP1 (adipocyte enhancer-binding protein 1), PGC1 (PPARγ-coactivator 1), and NF-κB (nuclear factor kappa-light-chain-enhancer of activated B).

Lipogenesis is the cell’s ability to synthesize fatty acids from other energetic sources, such as from glucose-derived acetyl-CoA. Fatty acids are synthesized from acetyl-CoA through the action of enzymes, such as acetyl-CoA carboxylase (ACC) and fatty acid synthase (FAS), with further processing by desaturases and/or elongases. The resulting fatty acids can be esterified to glycerol-3-phosphate or cholesterol to form triglycerides or cholesterol esters, respectively. These anabolic processes are shown in the simplified schematic of Figure 1.

Sterol regulatory element-binding proteins (SREBPs) are transcription factors that regulate lipogenesis via ACC and FAS, which are responsible for the first steps of de novo lipogenesis. Among the isoforms of SREBP, SREBP-1a is the most expressed in macrophages. SREBP-1a has been previously associated with both lipogenesis and phagocytosis in classically activated M1 macrophages [18,19]. The M1 macrophage response, induced by lipopolysaccharides (LPSs) via the Toll-like receptor 4 (TLR4) signaling pathway, results in increased lipogenesis and phagocytosis. However, when SREBP-1a was deleted in bone-marrow-derived macrophages, both lipogenesis and phagocytosis were reduced [18]. Additionally, SREBP-1 and the SREBP cleavage-activating protein (SCAP) were required to maintain phospholipids (phosphatidylcholine and phosphatidylethanolamine) in macrophage membranes [18]. The reduced phagocytic activity caused by the deleted SREBP-1 was restored when the medium was supplemented with exogenous fatty acids (oleic acid; C18:1) [18]. Consistently, both LPSs and palmitate (C16:0) increased the FAS activity in RAW 264.7 cells [1]. Taken together, lipogenesis via the SREBP signaling pathway is essential for the lipid composition and phagocytosis in macrophages.

Lipogenesis is also related to the production of cytokines induced by LPSs in M1 macrophages. LPSs and palmitate stimulated the production of TNF-α and IL-1β, but the increased cytokine levels were significantly reduced in FAS-knockout macrophages [1]. Furthermore, LPS-mediated IL-1β production was partially dependent on SREBP-1a [19]. SREBP-1a potentially acts as an upstream regulator of the NOD-like receptor family pyrin-domain-containing 1a (Nlrp1a), which contributes to IL-1β production via caspase-1 [19]. Another inflammatory caspase, caspase-11, was positively associated with SREBP activation. Caspase-11 is a protease activated by TLR4, and is part of the non-canonical activation of inflammasomes and pyroptosis, similar to caspase-1. Recent research has shown that caspase-11 interacts with SREBP processing through site-1 protease (S1P) to activate SREBP-1a [20]. In addition, LPS-induced SREBP-1a activation via S1P in macrophages was dependent on caspase-11 [20]. Therefore, the activation of SREBP-1a and lipogenesis play a role in the release of proinflammatory cytokines in M1 macrophages.

Chemicals that have an impact on lipid metabolism have the ability to influence the polarization of macrophages. An example of this is ursodeoxycholic acid, a bile acid that induces M2 polarization followed by a reduction in inflammation caused by excessive fat accumulation in ob/ob mice [21]. These effects of ursodeoxycholic acid were associated with the inhibition of lipogenesis and the enhancement of fatty acid oxidation. Similarly, an oleanolic acid derivative induced M2 polarization in obese mice by promoting fatty acid oxidation [22]. It is noteworthy that changes in macrophage polarization may not solely result from lipogenesis and/or fatty acid oxidation. The effects of these chemicals on other tissues can also contribute to macrophage polarization, which in turn may be linked to the overall changes in energy homeostasis.

### 3.2. Cholesterol Metabolism

Cholesterol is an important precursor to cell signaling molecules, and macrophages have an important role in the regulation of cholesterol levels in an individual. It is known that M2-like macrophages, foam cells, are involved in the uptake and transport of circulating cholesterol from vessels to excretory pathways. Moreover, cholesterol levels in macrophages, regulated by multiple factors, are associated with inflammatory responses. SREBPs, PPARs, and LXRs are some of the factors that regulate cholesterol metabolism, including the cholesterol efflux capacity in macrophages (Figure 1).

SREBPs promote cholesterol biosynthesis, and the SREBP-2 isoform is the one associated with cholesterol metabolism in macrophages [23,24]. SREBP-2 promotes targets of the mevalonate pathway and interferon response genes in TNF-activated macrophages [24]. miR-33, a microRNA involved in SREBP signaling, is also involved in the production of proinflammatory and anti-inflammatory genes in M1 and M2 macrophages, respectively [25]. Moreover, SREBP and miR-33 inhibit cholesterol efflux via the ATP-binding cassette transporter A1 (ABCA1) in macrophages [23,25]. The impact of SREBPs on cholesterol and inflammatory genes may potentially contribute to the progression of atherosclerosis, a process in which foam cells play a significant role, and other associated diseases. Indeed, the deletion of SCAP, which controls SREBP activity, caused changes in the cholesterol metabolism that inhibited cholesterol efflux and induced proinflammatory M1 polarization in adipose tissue macrophages [26].

Liver X receptors (LXRs) promote cholesterol efflux via transporters, such as ABCA1, and apolipoproteins [27]. Recently, a study showed that LXR regulates cholesterol and cytokine production with caveolin-1, a multifunctional membrane protein known to be required for phagocytosis, in response to LPSs [27,28]. LXR activation has been shown to increase IFN-γ and M1 macrophage polarization, which may explain the reduction in tumors [29]. However, the effects of LXR activation or inhibition may be dependent on the LXR isoform, macrophage type, and associated tissues [30,31].

PPARs are also transcription factors that act on cholesterol metabolism, even though PPARs are less abundant in macrophages than in other tissues, such as adipose tissue. The isoforms PPAR-α and PPAR-γ induce cholesterol efflux by upregulating ABCA1 expression via LXR-α in macrophages [32]. PPARγ inhibits the IFN-γ- and LPS-induced genes in macrophages, indicating that the activation of PPARγ by its agonist, rosiglitazone, has anti-inflammatory effects [3]. Consistently, rosiglitazone treatment inhibits the accumulation of cholesterol and cholesterol esters in LPS-treated macrophages [33]. LPSs downregulate PPAR-γ, PPAR-δ, and LXR-α in macrophages [3,4]. The effects of LPSs on cholesterol homeostasis, including the suppression of cholesterol efflux, are partially due to the decreased ABCA1 expression [3,4]. These suggest that LPSs and PPARs have opposite effects in macrophages. In LPS-treated macrophages, the adipocyte enhancer-binding protein 1 (AEBP1) is involved in inflammatory responses via NF-kB and cholesterol metabolism via PPAR-γ [4,34]. However, the inflammatory effects of LPSs and their effects on cholesterol metabolism are not exclusively dependent on the PPAR-γ/AEBP1 pathway. Other factors might contribute to the effects of PPARs on cholesterol metabolism, such as the retinoid X receptor (RXR). Previously, the activation of PPAR-γ, PPAR-α, and RXR inhibited the accumulation of cholesterol and cholesterol esters in macrophages induced by LPSs [33], but further studies are needed to understand the network of cholesterol control.

### 3.3. Fatty Acid β-Oxidation

**Figure 2:**
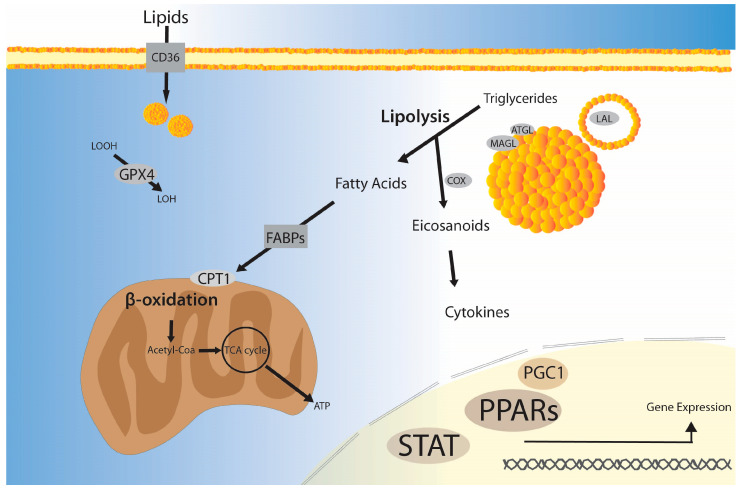
Schematic figure depicting lipolysis, fatty acid oxidation, and lipid uptake and transport. Lipids are taken up by CD36, a class B scavenger receptor, prior to incorporation on lipid droplets and organelles. The lipases ATGL (adipose triglyceride lipase), MAGL (monoacylglycerol lipase), and LAL (lysosomal acid lipase) are part of the catabolic reactions that break down triglycerides into fatty acids, releasing lipid mediators such as eicosanoids (e.g., prostaglandins) via the activity of COX (cyclooxygenase), for example. FABPs (fatty-acid-binding proteins) can transport free fatty acids into mitochondria for ATP production. CPT1 (carnitine palmitoyl transferase 1) is a key enzyme for β-oxidation that releases acetyl-CoA, which can be used in the tricarboxylic acid (TCA) cycle. GPX4 (glutathione peroxidase 4) is involved in the reduction of lipid peroxides (LOOH) into lipid alcohols (LOH), preventing ferroptosis and oxidative stress. The transcription factors and their associated co-factors, such as PPARs (proliferator-activated receptors), STA (signal transducer and activator of transcription), and PGC1 (PPARγ-coactivator 1), are part of the regulation of lipid-metabolism-related genes and their effects on macrophage functions.

Fatty acid β-oxidation (FAO) is a catabolic process that breaks down fatty acids into acetyl-CoA, which can be used in the mitochondrial tricarboxylic acid (TCA) cycle, as shown in the simplified schematic of Figure 2. Carnitine palmitoyl transferase 1 (CPT1) is an essential enzyme that transports cytoplasmic fatty acids into the mitochondria for subsequent FAO steps. The inhibition of CPT1 in macrophages has been associated with the progression of atherosclerosis by increasing the expression of CD36, which is involved in LDL uptake [35]. In addition, the levels of cholesterol esters in TNFα-treated macrophages are reduced when FAO is activated by PPARα, an upstream regulator of CPT1 [36]. Modulating FAO has been associated with overall fat/cholesterol levels and lipid uptake in macrophages, making FAO a potential target for the treatment of diseases [35,37].

FAO provides energy from lipids for macrophage functions, including M2 polarization, inflammatory responses, and phagocytosis. IL-4 increases FAO and fatty acid uptake, leading to a higher expression of CPT1, acyl-CoA dehydrogenases, and enoyl-CoA hydratases [38]. M2 macrophages most likely use fatty acids as their main energy source, while M1 macrophages use glucose; IFN-γ-/LPS-activated M1 macrophages have an increased glucose uptake, but not FAO [38]. However, FAO also contributes to the production of proinflammatory cytokines (IL-1β, TNF-α, IL-6, and IL-12), suggesting that FAO is also involved in the inflammatory response of M1 macrophages. The production of cytokines is dependent on CPT1 and inhibited by etomoxir [39,40,41]. Nevertheless, others have shown that inhibiting CPT1 with high doses of etomoxir also affects the overall CoA homeostasis, and other pathways might be involved in IL4-activated M2 macrophage polarization independently of CPT1 [42]. PPARγ-coactivator 1 (PGC1) and the signal transducer and activator of transcription (STAT) are part of the factors that control FAO and CPT1 [38,41,43]. Genetic approaches have also suggested the involvement of CPT1 in macrophage functions. A recent study suggests that CPT1 stimulates phagocytic activity in RAW264.7 macrophages. While a CPT1 knockdown through the adenovirus shRNA knockdown vector system caused a decrease in phagocytosis, macrophages with overexpressed CPT1 had higher phagocytic activity [44]. Overall, FAO is important for macrophage functions, although varying experimental conditions may yield different results.

### 3.4. Lipolysis

Adipose triglyceride lipase (ATGL) is one of the enzymes involved in the breakdown of triglycerides from lipid droplets in macrophages [45]. The inhibition of lipolysis via ATGL in macrophages increases lipid accumulation and impairs the production of cytokines (IL-6) induced by LPSs [45]. LPSs, which are known to increase fat accumulation, inhibit lipolysis by decreasing the ATGL expression in macrophages [46]. ATGL is involved in IL-6 production via PGE_2_, a lipid mediator [46]. Fatty acid uptake is increased in an attempt to compensate for the inhibited lipolysis in ATGL-null macrophages, but still, phagocytosis only occurs if there are available fatty acids for ATP production in macrophages [47]. Other lipases are also associated with macrophage functions. For example, monoacylglycerol lipase (MAGL), a lipase that breaks down monoacylglycerols into free fatty acids and glycerol, is involved in macrophage autophagy and inflammation [48]. The inhibition of lysosomal acid lipase (LAL) causes a decrease in M2 macrophage polarization and mitochondrial oxidative respiration, suggesting that lipolysis is also required for macrophage polarization [49]. Lipases are needed, in part, because they provide fatty acids that can be broken down by FAO, generating ATP (Figure 2). Moreover, the conversion of triglycerides into other lipid intermediates is related to other inflammatory signaling pathways. MAGL metabolizes 2-arachidonoylglycerol into arachidonic acid, a precursor of prostaglandins, by cannabinoid-receptor-dependent and -independent mechanisms [50]. MAGL is a potential target for cancer treatment, as others have shown that MAGL is associated with tumor progression and malignancy, which may be via the CB2 cannabinoid receptor [51,52].

### 3.5. Lipid Uptake and Transport

The CD36 receptor, known as glycoprotein IV, is a heavily glycosylated, 88 KDa receptor that was first discovered in platelets, but was later shown to be expressed in multiple cell types, including macrophages [53]. It belongs to the class B scavenger receptor group and is capable of binding to multiple ligands, including long-chain fatty acids (Figure 2) [54]. It is involved in the uptake of oxLDLs and oxidized lipids and the engulfment of apoptotic cells [55,56]. Its affinity for oxidized phosphatidylserine and, to a lesser extent, phosphatidylcholine facilitates the engulfment of apoptotic cells. It is assumed that unoxidized phosphatidylserine also plays a role in CD36 binding and the phagocytosis of apoptotic cells through externalization to the outer layer of plasma membranes. PPAR-γ has been shown to induce the upregulation of the CD36 receptor and promote macrophage differentiation as well as the enhanced uptake of oxLDLs [57]. Besides PPAR-γ, cytokines such as IL-10 and IL-4 are capable of modulating the differentiation of monocytes to macrophages [58]. While the increased binding of oxLDLs to the CD36 receptor in macrophages has been linked to atherosclerotic lesions, there are instances where the inhibition of CD36 leads to increased inflammation [59]. The CD36-mediated myelin clearance by macrophages and microglia was shown to reduce inflammation in an EAE model, highlighting the importance of CD36 in myelin debris clearance. From a genetic perspective, CD36 is encoded by an unusually polymorphic gene located on chromosome 7q11.2 [60]. Surprisingly, null alleles are present in over 20% in certain ethnic backgrounds without any significant impact on mortality rate [61,62]. Similar to oxLDLs, oxidized high-density lipoproteins (oxHDLs) increase CD36 palmitoylation and lipid uptake in macrophages [63]. The accumulation of oxidized lipids and cholesterol facilitates the eventual transformation of monocytes into foam cells [2]. Furthermore, the transformation to foam cells is accompanied by the augmentation of inflammatory cytokines such as IL-18, IL-8, and TNF-α. CD36’s central role in the foam cell transformation of monocytes has caused it to attract attention as a potential target for inhibition. Salvionolic acid B (SAB) has been shown to inhibit CD36, and its use in mice resulted in a reduction in visceral fat accumulation and an improvement in insulin resistance [2]. The use of omega-3 fatty acids had an analogous effect on THP-1 cells in terms of a reduction in foam cell transformation and inflammatory cytokines [64].

The transport of fatty acids within macrophages is important for overall lipid metabolism, affecting the immune properties of macrophages. Macrophage polarization is altered by fatty-acid-binding proteins (e.g., FABP-5), which are chaperones that transport lipids between organelles (Figure 2). A macrophage deficiency in FABP-5 stimulates fat accumulation via the PPAR-γ pathway, mainly affecting lipogenesis and FAO. Allergic asthma is aggravated by FABP-5 inhibition in vivo in an ovalbumin-induced allergic airway inflammation model. Interestingly, excessive oleic acid also aggravates allergic asthma by promoting M2 polarization, which is dependent on FABP-5 [65]. FABP-5 and inflammation are also linked in the context of hepatic inflammation [66]. These findings suggest that an unbalanced fatty acid homeostasis disrupts macrophage metabolism, subsequently impacting the surrounding tissues.

## 4. Macrophages and Cancer

Even though macrophages are a major player in innate immunity and one of the three professional antigen-presenting cells (APCs) of the immune system, in many instances, they support the growth of tumors. Tumor-associated macrophages (TAMs) are associated with tumor metastasis, immune suppression, and an overall poor prognosis. The expression of programed cell death protein 1 (PD-1) on TAMs has been shown to prevent phagocytosis and tumor immunity [67]. Even though PD-1 and programed cell death ligand 1 (PD-L1) interference using monoclonal antibodies is a clinically proven strategy for activating T-cells, it has recently been shown to be relevant to TAMs. The tumor microenvironment of breast and endometrial cancers has been shown to be capable of reprograming the transcriptomic profile of TAMs [68]. Of particular significance was the expression of CCL8 and SIGLEC1 in TAMs, which was associated with a shorter cancer-free survival rate. Lipids can play an important role in altering TAMs into a more protumor phenotype. An enhanced expression of the CD36 receptor and lipid were observed in TAMs [69]. The switch from glycolysis to FAO along with an increase in ROS played a key role in the polarization of TAMs. At the transcriptional level, STAT6 activation was mediated through the phosphorylation of Janus kinase 1 (JAK1) and the dephosphorylation of SHP1. Hedgehog (Hh) signaling was shown to influence the metabolism and polarization of mammary TAMs [70]. Lipid metabolism mediated via Hh signaling could be modulated using the inhibitor vismodegib. Specifically, Hh inhibited TAMs from switching from lipid metabolism to glycolysis. The metabolic change was associated with a transformation of the M2 phenotype to one that is typical of M1 macrophages. Lipid oxidation is a common hallmark of M2 macrophages. The cytokine IL-4 produced by Th2 cells supports protein kinase RNA-like ER kinase (PERK) signaling [71]. PERK signaling supports lipid oxidation and, thus, an M2 immunosuppressive phenotype. The trained immunity of metastatic macrophages using whole beta-glucan particles (WGPs) induced a reduction in metastasis in multiple mouse models [72]. The trained immunity was attributed to sphingosine-1-phosphate. The inhibition of the synthetic pathway of sphingosine-1-phosphate led to a reversal in anti-metastatic properties. Apolipoprotein E (ApoE) mediates the infiltration of TAMs in pancreatic ductal adenocarcinoma [73]. It induces the immunosuppression of TAMs in pancreatic cancer via the production of chemokine ligand 1 (CXCL1). In some instances, a related immune cell population has an indirect effect on macrophages in the tumor microenvironment. T-regs reduce IFN-γ production in CD8+ cells, which has a direct effect on SREBP-1 [74]. The effect of SREBP-1 on fatty acid synthesis supports the M2-like phenotype of macrophages and the overall immunosuppressive character of TAMs. The inhibition of SREBP-1 enhanced the immune checkpoint blockade, once again highlighting the role of lipids in macrophages within tumors. Dietary omega-3 fatty acids had an anti-M2 effect on macrophages in a model of a castrate-resistant prostate in comparison to omega-6 fatty acids [75]. It is evident that macrophages are susceptible to manipulation and reprogramming in the tumor microenvironment. Lipids are crucial participants in this manipulation and reprogramming in various ways that range from basic metabolism to specific signaling pathways.

## 5. Macrophages and Cyclooxygenase Inhibitors

The arachidonic acid (AA) metabolic pathway was among the first to be elucidated. Pharmacologic manipulation of this pathway has been very successful, particularly in terms of inflammation, pain, fever, and coagulation [76]. The products of the AA metabolic pathway are diverse and complex. PGE_2_, prostacyclin I2, thromboxane, and leukotrienes are among the best-understood metabolites of AA, with properties that can easily be manipulated using widely available pharmacologic agents known as cyclooxygenase (COX) inhibitors and leukotriene receptor antagonists [77]. The impact of the AA metabolites on the immune system is profound, and the pharmaceutical agents used for decades now have been very effective at alleviating the symptoms of various medical conditions [78]. Given the anti-inflammatory nature of COX inhibitors, it is not a surprise that macrophages are amenable to inflammatory regulation in the presence of these drugs. Celecoxib is the only COX-2 inhibitor currently being used for inflammation and pain. It has a distinct effect on macrophages via CD36 and the class E receptor of oxidized low-density lipoprotein (LOX1) [79]. In the presence of high concentrations of celecoxib, THP-1 cells and peripheral blood mononuclear cells had a significantly reduced expression of 27-hydroxylase and ABCA1. The overall effect was a reduction in cholesterol outflow from macrophages and, thus, likely a positive outcome with respect to atherosclerosis. A low dose of celecoxib showed similar anti-inflammatory results in a model involving neuroinflammation of the sciatic nerve in rats [80]. A reduction in monocyte infiltration at the site of the injury was observed along with COX-2 and PGE_2_. In a cancer study, COX-2 inhibition using flunixin meglumine inhibited the M2 polarization of macrophages initiated in the hypoxic tumor environment [81]. The positive influence of COX inhibition on macrophage polarization has attracted attention in the field of oncology as a therapeutic strategy. Aspirin, which is considered a non-selective COX inhibitor, has the ability to decrease the phagocytosis and immunogenicity of murine peritoneal macrophages [82]. The use of aspirin to control inflammation is common. However, it is well documented that it can cause gastrointestinal complications [83]. Ibuprofen, another widely used nonselective COX inhibitor, also has a polarization influence on macrophages and an overall anti-tumor benefit [84]. Using a syngeneic (D2A1) orthotopic Balb-c mouse model, ibuprofen reduced the infiltration of immature monocytes into tumors implanted in the mammary involution. Despite the anti-inflammatory character of nonsteroidal anti-inflammatory drugs, some studies show that the use of COX inhibitors decreases the levels of anti-inflammatory cytokines such as IL-10 [85]. It is conceivable that the use of COX inhibitors does not simply reduce inflammation, but rather modulates inflammation in ways that are clinically beneficial.

## 6. Macrophages and Ferroptosis

Ferroptosis is an intriguing cell death pathway that is distinct from the classical apoptotic pathways [86]. Lipid peroxidation, an imbalance of glutathione and iron, plays a central role in ferroptosis [87]. The term ferroptosis was first introduced by Dixon et al. due to the exclusive reliance on iron and not any other metal for the initiation of this death pathway. Erastin, a small molecule, induces ferroptosis while ferrostatin-1 acts as a potent inhibitor of ferroptosis in cancer cells. Unsurprisingly, glutathione behaves as a buffering system, and its depletion leads to ferroptosis [88]. Interestingly, the conditional deletion of glutathione peroxidase 4 (GPX4), an enzyme that catalyzes lipid peroxides utilizing glutathione, does not have a significant impact on the inflammatory properties of macrophages [89]. On the other hand, ferroptosis was induced when GPX4 was deleted in M2 macrophages, suggesting that ferroptosis and the requirement for GPX4 may be dependent on macrophage polarization. Lipid peroxides can be reduced by GPX4 (Figure 2), and reduced levels of lipid peroxides cause a decrease in lipoxygenase (LOX) activity [90]. Ferrous iron is a co-factor for LOXs, which are involved in the oxidation of fatty acids, especially polyunsaturated fatty acids [90]. Indeed, Fe_3_O_4_ nanoparticles reduce macrophage viability and promote the M1 phenotype [91]. The expression of p53, GPX4, and the transferrin receptor (TFR) were upregulated in macrophages stimulated with Fe_3_O_4_ nanoparticles and was similar to that resulting from treatment with erastin. TFR is involved in the uptake of Fe^3+^ into cells, which, in excess, can induce ferroptosis via the production of lipid peroxides [90]. The tumor microenvironment often exhibits a bias towards the M2 phenotype, which promotes immunosuppression [92]. Dihydroartemisinin, a chemotherapeutic agent, supports the M1 phenotype by inducing ferroptosis in macrophages, both in vitro and in vivo, by the downregulation of GPX4. NF-κB activation may be responsible for the shift towards the M1 phenotype. Ferroptosis had a similar effect on macrophages in a breast cancer model, where M2 suppression resulted in a reduction in the migration and invasion of breast cancer cells [93]. However, there are instances where ferroptosis and its byproducts encourage an M2-like phenotype. This is the case with KRAS (Kirsten Ras oncogene), one of the most common mutated oncogenes in cancer. It has been shown to play a critical role in pancreatic-tumor-associated macrophage polarization [94]. The release of KRAS by cancer cells undergoing ferroptosis packaged in exosomes and absorbed by macrophages leads to an M2-like phenotype. The levels of KRAS in the macrophages of patients with pancreatic cancer are correlated with a low survival rate. In hepatocellular carcinoma (HCC), the inhibition of ferroptosis suppressor protein 1 (FSP1) increases immune cell infiltration, including macrophages [95]. Moreover, the inhibition of FSP1 in conjunction with immunotherapies suppresses HCC progression via the augmentation of ferroptosis and immune activation. Overall, ferroptosis manipulation is an attractive strategy in the fight against cancer. What remains a challenge is the targeting of ferroptosis towards malignant cells and the reprograming of macrophages to an anti-tumor phenotype.

## 7. Conclusions

Several studies have proven that lipids and their metabolites influence macrophage polarization, their anti-/proinflammatory properties, and phagocytosis. SREBPs, PPARs, LXRs, and other lipid-metabolism-related factors are also important for macrophage functions. Moreover, the manipulation of these factors can be part of treatment strategies in macrophage-related diseases, such as atherosclerosis and cancer. There are various ways to alter the energy sources of macrophages and functions by targeting lipid metabolism. However, the implementation of therapies may face limitations as compensatory and overlapping mechanisms within lipid metabolism come into play, highlighting the need for further research.

## Data Availability

No new data were created or analyzed in this study. Data sharing is not applicable to this article.
